# Application of Four Genotyping Methods to Mycoplasma bovis Isolates Derived from Western Canadian Feedlot Cattle

**DOI:** 10.1128/JCM.00044-21

**Published:** 2021-06-18

**Authors:** Andrea Kinnear, Matthew Waldner, Tim A. McAllister, Rahat Zaheer, Karen Register, Murray Jelinski

**Affiliations:** a Western College of Veterinary Medicine, University of Saskatchewan, Saskatoon, Saskatchewan, Canada; b Lethbridge Research and Development Centre, Agriculture and Agri-Food Canada, Lethbridge, Alberta, Canada; c Ruminant Diseases and Immunology Research Unit, USDA Agricultural Research Service, National Animal Disease Center, Ames, Iowa, USA; University of Tennessee at Knoxville

**Keywords:** beef, feedlot, genotyping, *Mycoplasma*

## Abstract

Mycoplasma bovis is a significant pathogen of feedlot cattle, responsible for chronic pneumonia and polyarthritis syndrome (CPPS). M. bovis isolates (*n *= 129) were used to compare four methods of phylogenetic analysis and to determine if the isolates’ genotypes were associated with phenotypes. Metadata included the health status of the animal from which an isolate was derived (healthy, diseased, or dead), anatomical location (nasopharynx, lung, or joint), feedlot, and production year (2006 to 2018). Four *in silico* phylogenetic typing methods were used: multilocus sequence typing (MLST), core genome MLST (cgMLST), core genome single nucleotide variant (cgSNV) analysis, and whole-genome SNV (wgSNV) analysis. Using Simpson’s diversity index (*D*) as a proxy for resolution, MLST had the lowest resolution (*D* = 0.932); cgSNV (*D* = 0.984) and cgMLST (*D* = 0.987) generated comparable results; and wgSNV (*D* = 1.000) provided the highest resolution. Visual inspection of the minimum spanning trees found that the memberships of the clonal complexes and clades had similar structural appearances. Although MLST had the lowest resolution, this methodology was intuitive and easy to apply, and the PubMLST database facilitates the comparison of sequence types across studies. The cg methods had higher resolution than MLST, and the graphical interface software was user-friendly for nonbioinformaticians, but the proprietary software is relatively expensive. The wgSNV approach was the most robust for processing poor-quality sequence data while offering the highest resolution; however, application of its software requires specialized training. None of the four methods could associate genotypes with phenotypes.

## INTRODUCTION

Mycoplasma bovis is associated with a plethora of diseases in cattle. Reviews on this subject commonly emphasize its role in chronic pneumonia, mastitis, and arthritis (1–3). While the incidence of M. bovis mastitis in Canada is relatively low (4, 5), it is considered an emerging disease of dairy cattle (6). The incursion of M. bovis into New Zealand and that country’s considerable efforts to eradicate this organism underscore its potential to cause significant economic losses to the dairy industry. In the feedlot industry, M. bovis is associated with bovine respiratory disease (BRD) and chronic pneumonia and polyarthritis syndrome (CPPS) (7). However, its role in the pathogenesis of BRD has been equivocal. In the early 1990s, it was hypothesized that M. bovis was unlikely to be a primary pathogen but potentiated the pathogenesis of other bacterial BRD agents (8). It is now considered a primary pathogen of pneumonia in preweaned calves (9, 10) and an opportunistic pathogen of BRD in feedlot cattle (1, 2, 7). Cattle are frequently asymptomatic carriers; the organism is recovered from the nasal passages of healthy cattle (11–13). It is hypothesized that stressors such as transport, commingling, and adverse climatic conditions trigger a stress response, resulting in elevated levels of glucocorticoids that may impair the immune response, allowing for increased bacterial shedding and clinical disease (14).

A number of molecular techniques have been used for typing M. bovis isolates. The formative techniques included pulsed-field gel electrophoresis (PFGE) (15–17), random amplified polymorphic DNA (RAPD) (15, 18), and amplified fragment length polymorphism (AFLP) (15, 19). These methods have been largely replaced by PCR-based methods such as multilocus sequence typing (MLST) and multiple-locus variable-number tandem-repeat analysis (MLVA) (20–22). While MLVA has greater discriminatory power than MLST (21), the latter method is an unambiguous, reproducible, and scalable procedure for characterizing isolates of bacterial species by using a universally acceptable schema (23) (https://pubmlst.org/). These attributes make MLST well suited for epidemiological studies spanning multiple research laboratories and for the comparison of isolates over time (21, 24–26).

Core genome MLST (cgMLST) is an extension of MLST but provides higher resolution. Whereas a typical MLST scheme uses the alleles of seven housekeeping genes, cgMLST analyzes alleles from hundreds of genes, an approach facilitated by whole-genome sequencing (WGS). cgMLST has been used for typing poultry mycoplasmas (27), investigating outbreaks of M. bovis in dairy cattle (28, 29), and examining the genetic relatedness and evolution of isolates obtained from cattle in Denmark and neighboring countries (30). The authors of the Denmark study noted that the cgMLST and WGS typing techniques offered greater discriminatory power than MLST and hence may become the new standard in phylogenetic typing. However, these methods do have disadvantages, namely, cost, time, and the need for technical expertise for conducting the analyses and interpreting the results.

A further progression into higher-resolution strain typing involves genome-wide comparisons of single nucleotide variants (SNVs). This method can be applied to SNVs in the core genome (cgSNVs) or the whole genome (wgSNVs). Using WGS, Australian researchers concluded that 75 M. bovis isolates collected between 2006 and 2015 were of the same lineage, suggesting few, if any, incursions of new strains over the study duration. Similarly, an Israeli study used cgSNV analysis to evaluate the genomic diversity of M. bovis isolates from mastitis cases between 1994 and 2017 and compared these to BRD isolates from local feedlot cattle and from calves imported from Europe and Australia (31). There was a clear genetic distinction between the isolates from Europe and Australia, and a dominant genotype was associated with mastitis. wgSNV analysis has also been used to compare the relatedness of 250 M. bovis isolates originating from seven countries (32). These isolates formed six clades, with U.S. isolates exhibiting the greatest genetic diversity but also clustering with Canadian isolates.

The objectives of this study were, first, to assess the level of concordance between four different molecular genotyping methods (*in silico* MLST, cgMLST, cgSNV, and wgSNV) by using a data set of 129 M. bovis isolates and, second, to determine if one or more methods could resolve genotypic differences among isolates derived from cattle of different health statuses (healthy, sick, and dead cattle), from different anatomical locations (nasopharynges, lungs, and joints), from different feedlots, and over a 12-year period (2006 to 2018).

## MATERIALS AND METHODS

### Sample collection.

A series of cross-sectional studies, spanning the years 2006 to 2018, provided 129 M. bovis isolates, which were recovered from the nasopharynges, lungs, and joints of feedlot cattle, as described previously (33). Five isolates were recovered from cattle imported from Idaho, USA, while all others were from cattle resident in western Canada. The deep nasopharyngeal (DNP) swabs were obtained from healthy and morbid cattle. Each animal’s health status was determined by the timing of the disease and the presentation of clinical signs. BRD is the most common disease of feedlot cattle, and cases peak within 21 days after arrival at the feedlot. Thus, a putative BRD diagnosis was based on the timing of the disease and a constellation of the clinical signs consistent with this disease (i.e., fever, depression, nasal discharge, dyspnea). Health status was determined by trained feedlot personnel. The DNP swabs from live cattle were obtained in accordance with Animal Use Protocols (no. 20070023 and 20170021) approved by the University of Saskatchewan’s Animal Research Ethics Board and Lethbridge Research Centre’s Animal Care Committee (no. 1641). Lung and joint samples were obtained at the time of postmortem examination from cattle with gross pathological findings consistent with caseonecrotic bronchopneumonia and, in some instances, concurrent septic arthritis.

### Culture and isolation.

Small changes in media and isolation methods occurred over the 12-year period; the isolates recovered in 2006 to 2008 were cultured in Hayflick medium (prepared in-house) (34), and all subsequent isolates were cultured with pleuropneumonia-like organism (PPLO) broth and agar (BD Difco, Fisher Scientific, Waltham, MA, USA). The PPLO media were supplemented with 10 g/liter yeast extract (BD Diagnostic Systems, Fisher Scientific) and 20% horse serum (Invitrogen, Fisher Scientific), as described previously (33). Supplemented media also contained 0.05% thallium(I) acetate, 500 U/ml penicillin G, and/or 0.5% sodium pyruvate (Sigma-Aldrich, St. Louis, MO, USA).

DNP samples and swabs of fresh-cut tissues were used to inoculate PPLO starter cultures. Cultures were serially filtered through 0.45- and 0.20-μm-pore-size filters (Basix; VWR International, Radnor, PA, USA) to remove other bacteria, such as coinfecting BRD pathogens (0.45 μm), and to select for *Mycoplasma* spp. (0.20 μm). Filtrates were inoculated into supplemented PPLO broth and were grown under a 5% CO_2_ atmosphere with 75% humidity at 37°C. Culture growth was visualized by agitating the culture tube to elicit a perceptible mass of organisms at the bottom of the tube. Cultures with visible growth were subcultured onto PPLO agar and incubated for 3 to 6 days. Single colonies exhibiting a “fried-egg” morphology were picked, plated on PPLO agar, and incubated for 72 h. One to three individual colonies per culture were used to inoculate separate aliquots of PPLO broth. After 48 h, each culture was stored in PPLO medium with 20% (vol/vol) glycerol at –80°C. A single culture was chosen to inoculate the PPLO medium for DNA extraction.

### DNA extraction and identification.

Isolates were grown in PPLO medium for 48 h, and genomic DNA was extracted using the GenElute Bacterial Genomic DNA kit (Sigma-Aldrich) according to the manufacturer’s instructions, except that the final elution buffer was replaced with 10 mM Tris (pH 8.5). The extracted genomic DNA was assessed for quality using gel electrophoresis and was quantified fluorometrically using a Qubit analyzer (Thermo Fisher Scientific). The isolation of high-molecular-weight DNA with a yield of ≥1 ng/μl was sufficient to proceed. Cultures were confirmed as M. bovis by using a species-specific PCR assay targeting the *uvrC* gene (35) and by sequencing the V3–V4 16S rRNA gene (36). The 16S rRNA amplicon was purified using a QIAquick PCR kit (Qiagen, Venlo, Netherlands) and was submitted for Sanger sequencing (Macrogen, Seoul, South Korea). Forward and reverse sequences were assembled and edited using the Staden package (version 1.6-r; http://staden.sourceforge.net/) and were compared to the National Center for Biotechnology Information (NCBI) nonredundant (nr) nucleotide database using BLASTn run with default settings. The species was assigned based on the highest match identity (37).

### Whole-genome sequencing and assembly.

Sequencing libraries of genomic DNA were prepared using an Illumina Nextera XT DNA library preparation kit (Illumina Inc., San Diego, CA, USA) and were sequenced on an Illumina MiSeq platform using the MiSeq v2 reagent kit to generate 250-bp paired-end reads. Genomes were assembled for the MLST, cgMLST, and cgSNV methods using Ridom SeqSphere+ in pipeline mode (38). Raw paired-end reads were imported and preprocessed by down-sampling to 180× coverage and trimming at the 5′ and 3′ ends until an average quality of 30 in a window of 20 bases was achieved. Reads were assembled using SKESA (39). Genome assembly for the wgSNV method was performed using Trimmomatic, v0.39 (40), for read trimming and SPAdes, v3.14.1 (41), for *de novo* assembly of the contigs. Trimmomatic settings were as follows: sliding window, 5:15; leading, 5; trailing, 5; minlen, 25. SPAdes was run with settings -careful and -k 127. Contigs with <1,000 nucleotides were removed from the analysis.

### Genotyping methods.

*De novo* assemblies were used to assign allelic profiles and sequence types (STs) as per the PubMLST reference method (https://pubmlst.org/organisms/mycoplasma-bovis/). The M. bovis PG45 reference genome (GenBank accession no. NC_014760.1) was included in each genotyping method with the 129 isolates. The MLST scheme included alleles of the following genes: *dnaA*, *gltX*, *gpsA*, *gyrB*, *pta-2*, *tdk*, and *tkt* (25; version update, 15 March 2021).

The same *de novo* assemblies were used to develop an *ad hoc* cgMLST scheme using Ridom Seqsphere+ (version 6.0.2) (38). The reference strain M. bovis PG45 (GenBank accession no. NC_014760.1) was used as the seed genome with the following criteria: minimum length, ≥50 bases; start and end codons on each end of the gene; no multiple copies of genes with a BLAST overlap of ≥100 bp with an identity of ≥90%; and no overlap with genes of >4 bases. Genes identified in the seed genome were queried against the following 10 penetration genomes in order to identify genes for inclusion in the final *ad hoc* cgMLST scheme: M. bovis Hubei-1 (GenBank accession no. NC_015725.1), HB0801 (NC_018077.1), CQ-W70 (NZ_CP005933.1), NM2012 (NZ_CP011348.1), HB0801-P115 (NZ_CP007589.1), 08M (NZ_CP019639.1), Ningxia-1 (NZ_CP023663.1), JF4278 (NZ_LT578453.1), 16M (NZ_CP038861.1), and XBY01 (NZ_CP045797.1). Penetration genomes were queried using BLAST (version 2.2.12) and were required to have equivalent targets that met the BLAST hit overlap of 100% with an identity of ≥90% in all query genomes. The following criteria were used: word size, 11; mismatch penalty, –1; match reward, 1; gap open costs, 5; and gap extension costs, 2. Targets were also required to have a single stop codon at the end of the gene in >80% of penetration query genomes. The resulting cgMLST scheme consisted of 506 genes (loci) and covered 55.1% of the M. bovis PG45 genome.

The distance matrix used for cgMLST phylogenetic analysis omitted genomes missing >10% of distance columns and removed columns with missing values. This resulted in isolates being typed based on the alleles of 296 loci.

SNVs for the cgSNV method were determined from 283 gene targets (loci) of the M. bovis PG45 reference genome (NC_014760.1). Comparison of all genomes to these targets yielded 6,408 SNV positions, which were filtered to 3,925 SNVs in 283 loci by including only substituted SNV positions (hiding insertions/deletions) and having no neighboring SNV positions in a window of 10 bases. SNVs for the wgSNV method were identified within the 130 genomes by kSNP, v3.1 (42), which yielded an SNV matrix file. The settings used for kSNP were -core and -k 31. The SNV matrix contained 14,383 SNVs across all genomes.

Simpson’s diversity index (43) compared the discriminatory power of each strain-typing method, based on the clustering of isolates within the minimum spanning trees (MSTs) for the MLST, cgMLST, and cgSNV methods. The wgSNV method did not cluster any two of the isolates into a single type. Therefore, each isolate was classified as a unique type, as defined by a unique genotype for each isolate.

### Data presentation.

Neighbor joining (NJ) trees were created with iTOL (44) and MSTs with Ridom SeqSphere+. A maximum-likelihood tree of the wgSNV matrix was generated with the Tamura-Nei substitution model, using uniform rates and the nearest-neighbor-interchange heuristic method with MEGA X, v10.1.1 (45). The tree was visualized with iTOL and GrapeTree (46). Isolates were grouped into clonal complexes (CC) or clades (C), as appropriate. A CC was defined as a group of isolates with STs that differed by no more than two alleles from at least one other ST in the group. A singleton was a clonal group that differed from all other STs by at least three alleles. A clade was defined as a group of strains with a common biological ancestor. The CC were assigned by MLST analyses, while clades comprised the MSTs of the cgMLST, cgSNV, and wgSNV analyses. These MSTs were determined by visual assignment based on the root of a tree or an ST central to the tree that served as a common ancestor.

### Data availability.

The raw paired-end reads generated in this study are available from the Sequence Read Archive (SRA) under BioProject accession no. PRJNA642970 and PRJNA708306.

## RESULTS

### Isolates and assembly.

M. bovis isolates (*n *= 129) spanning 12 production years (2006 to 2018) and 21 feedlots (A to U) were recovered from 98 individual feedlot cattle (see Table S1 in the supplemental material). All 21 feedlots were located in western Canada; 45.0% (*n *= 58) of the isolates originated from two feedlots (N, Q), each with a capacity of >20,000 head. Isolates were recovered from the nasopharynges (*n *= 49), lungs (*n *= 45), and joints (*n *= 35). The numbers of isolates by animal health status were as follows: 82 from dead cattle, 32 from healthy cattle, and 15 from diseased cattle. DNP isolates (*n *= 49) were recovered from healthy (*n *= 32), diseased (*n *= 15), and dead (*n *= 2) cattle. All isolates (*n *= 129) underwent WGS and *de novo* assembly with the following criteria: mean *N*_50_, 18,448 (range, 997 to 32,908); contig count, 186 (range, 79 to 797); coverage, 84 (range, 12 to 177); approximate completed genome size, 90% (range, 60% to 100%) relative to the PG45 reference genome.

### MLST.

Of 130 genomes (129 isolates plus the PG45 reference genome), 126 were assigned an existing ST. Four isolates could not be typed due to a missing allele(s) and are designated as ST “Unknown” (one also had a novel allele at the *pta-2* locus), which is perhaps a reflection of low-quality assemblies (*N*_50_, 997 to 2,494; contig count, >500; coverage, 12 to 51; approximate genome size, ≤80%). The MLST scheme typed the 125 isolates into 24 known STs and 6 newly identified STs (ST149 to ST154) (Table 1). The PG45 reference genome included in the analysis was assigned ST12, as expected. Simpson’s diversity index was 0.932, indicating reasonably strong separation of isolates. Two previously uploaded isolates that had been assembled using SPAdes (41) had MLST STs differing from those assigned by SKESA (39) and Ridom SeqSphere+. Two of these were within the set of four isolates that had missing alleles. These differences are likely due to low-quality isolates and alternate assembly processes.

**TABLE 1:** Numbers of MLST sequence types of Mycoplasma bovis isolates by production year and health status^*a*^

Sequence type	No. of isolates											
	Production yr								Health status			Total
	2006	2007	2008	2014	2015	2016	2017	2018	Healthy	Diseased	Dead	
2		2				1				1	2	3
14							1		1			1
21	1	4					2		2	3	2	7
24		12			1				5	6	2	13
27								3			3	3
40		5							3	2		5
42	3	2		1	1				3	2	2	7
43								1	1			1
44							9				9	9
45							3	2	4		1	5
48		1							1			1
52		1				3					4	4
60				1		13	1	8			23	23
61						1					1	1
62		1							1			1
65						6	1		1		6	7
66							2		2			2
67							12				12	12
70		1			2					1	2	3
75			1								1	1
76				2							2	2
77						2					2	2
79						1					1	1
80						3					3	3
149								1			1	1
150								1	1			1
151	1								1			1
152							1		1			1
153								2	2			2
154							2		2			2
Total	5	29	1	4	4	30	34	18	31	15	79	125

a A total of 125 M. bovis isolates were tested. PG45 (ST12) is excluded from this table.

The most prevalent STs were ST60 (23 of 130 isolates [17.69%]), ST24 (13 of 130 [10.00%]), and ST67 (12 of 130 [9.23%]). These prevalences were higher than those reported in the PubMLST database (https://pubmlst.org/organisms/mycoplasma-bovis/, accessed 12 March 2021): 12 of 1,139 isolates (1.05%) in ST60, 9 of 1,139 isolates (0.79%) in ST24, and 9 of 1,139 isolates (0.79%) in ST67. Among the 23 ST60 isolates, 12 had been used in another, unrelated study (25) and are represented in the PubMLST database, but under isolate identifiers (see Table S1). The STs with the highest frequencies in the PubMLST database were ST52 (127 of 1,139 isolates [11.15%]), ST62 (71 of 1,139 isolates [6.23%]), and ST21 (31 of 1,139 isolates [2.72%]), all of which are frequently identified in North America but were infrequent in the current study.

Isolates (*n *= 35) recovered from feedlot N between the years 2016 and 2018 were represented by nine STs, of which ST60 (*n *= 10 [28.6%]) was the most prevalent in all years. The 22 isolates recovered from feedlot Q in 2007 were categorized into six STs; 12 (54.5%) were in ST24. Two STs (ST2 and ST21) were of particular interest because they were separated in time and space. ST2 was isolated from feedlot Q in 2007 and from feedlot N in 2016, while ST21 was recovered from feedlot Q in 2007 and from feedlot N in 2017. These feedlots were separated by a distance of approximately 500 km. Five isolates were derived from cattle imported from the northern United States.

**FIG 1:**
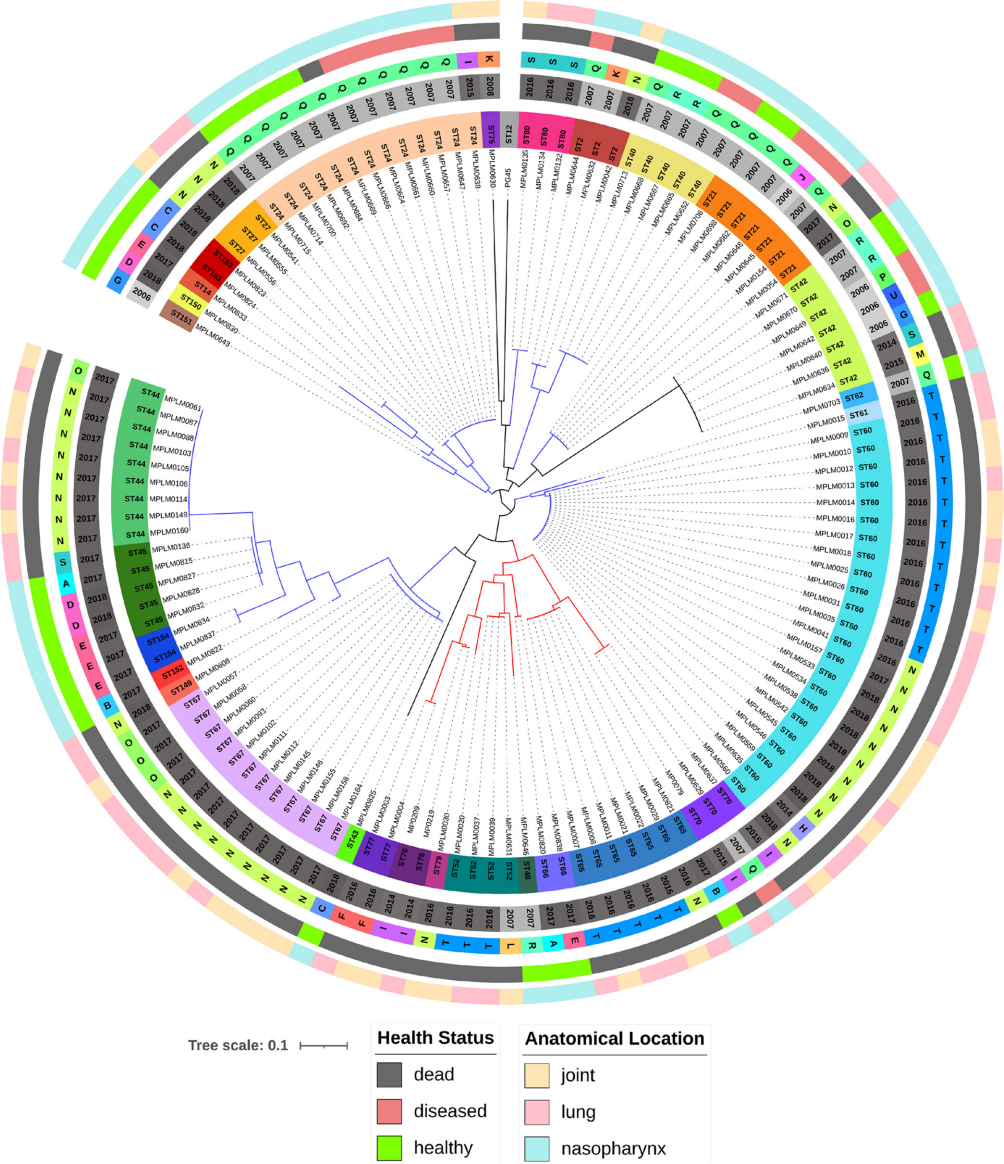
MLST neighbor joining tree of 126 Mycoplasma bovis isolates typed by MLST. The rings (starting from the innermost) contain information on isolate identifiers, MLST sequence type (ST), production year, feedlot (A to U), anatomical sampling location, and animal health status at the time of sampling. Two clonal complexes are depicted with red (CC1) and blue (CC2) branch lines. Four singletons (ST12, ST42, ST43, ST75) are depicted as black branch lines.

Isolates clustered into two clonal complexes (CC1, CC2) and as four singletons, including the PG45 reference strain (ST12, ST42, ST43, ST75) (Fig. 1). The ST52 and ST60 isolates formed the foci of the CC in the MLST minimum spanning tree (Fig. S1). Two isolates (MPLM0830, MPLM0608) from 2018 had allelic combinations that had not been described previously. Five STs persisted within the western Canadian cattle population for many years: ST2 (2007 to 2016), ST21 (2006 to 2017), ST24 (2007 to 2015), ST42 (2006 to 2015), and ST70 (2007 to 2015). Three of these STs (ST2, ST21, ST24) were grouped in CC2, whereas ST70 was grouped with CC1 and ST42 was a singleton.

The data set included 31 pairs of lung-joint samples obtained from individual animals; for 28 lung-joint pairs, both isolates were successfully typed using MLST. In 18 (64.3%) instances, the ST recovered from the lung matched the ST found in the joint of the same animal.

### cgMLST and cgSNV analyses.

**FIG 2:**
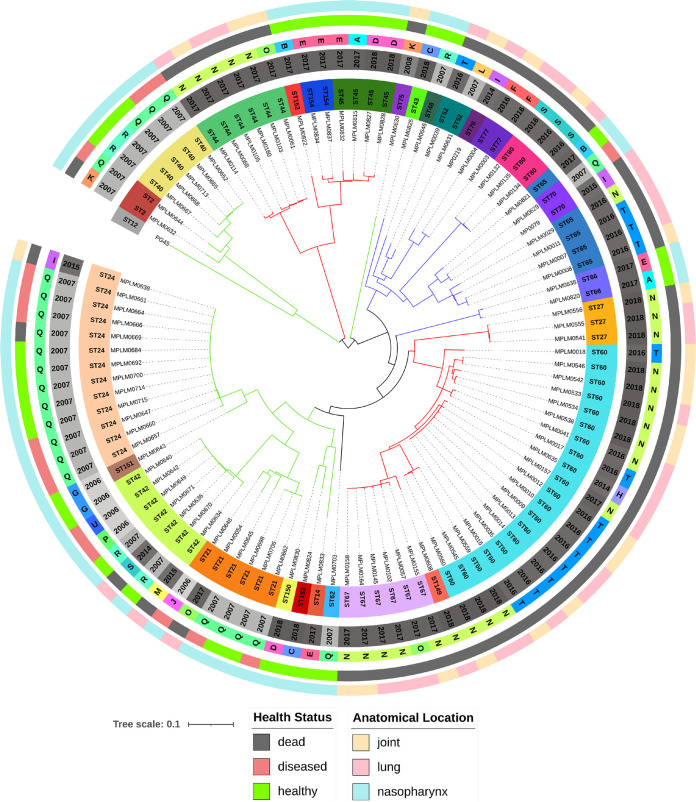
cgMLST neighbor joining tree of 102 Mycoplasma bovis genomes based on alleles at 296 core genome loci. The rings (starting from the innermost) contain information on isolate identifiers, MLST sequence type (ST), production year, feedlot (A to U), anatomical sampling location, and animal health status at the time of sampling. The branches are colored in accordance with the three clades (C1, red; C2, blue; C3, green) identified in Fig. S2, highlighting the differences between the tree construction methods.

**FIG 3:**
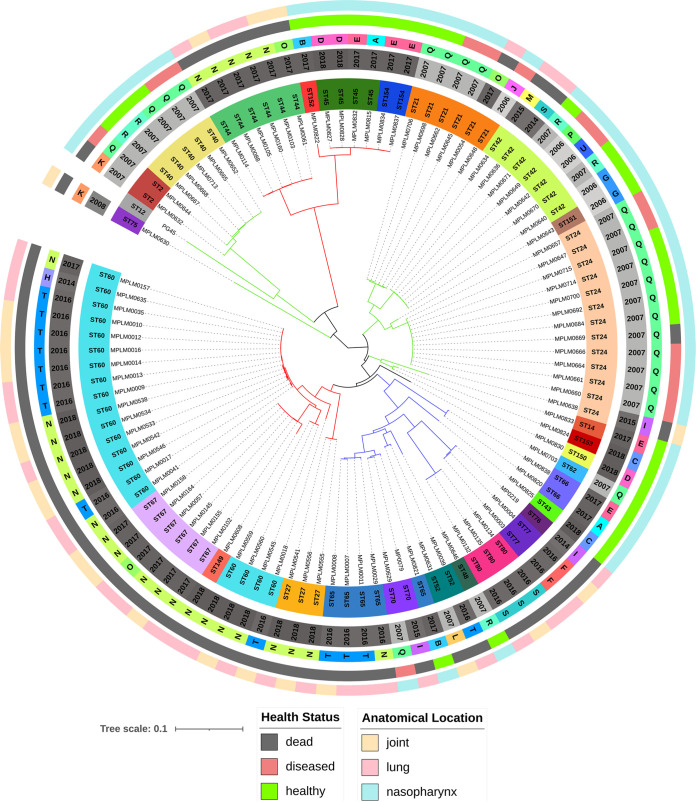
cgSNV neighbor joining tree of 102 Mycoplasma bovis isolates based on 283 core genome loci (3,925 SNVs). The rings (starting from the innermost) contain information on isolate identifiers, MLST sequence type (ST), production year, feedlot (A to U), anatomical sampling location, and animal health status at the time of sampling. The branches are colored to match the clades (C1, red; C2, blue; C3, green) identified in Fig. S3, highlighting the differences between the tree construction methods.

A total of 102 genomes (101 isolates and PG45) were typed by the cgMLST (Fig. 2; Fig. S2) and cgSNV (Fig. 3; Fig. S3) methods. Figures S2 and S3 provide the MSTs of the isolates, with three clades (C1, C2, C3) branching from a single focus consisting of an isolate with MLST ST62. Isolates from these clades tended to cluster together in the neighbor joining (NJ) tree, but small subclades (Fig. 2 and 3) branched from the root and contained isolate-specific clades in the MSTs. Both cg analyses grouped the five STs discussed above (ST2, ST21, ST24, ST42, and ST70) into two clades: ST2, ST21, ST24, and ST42 were grouped in C3, and ST70 was allocated to C2 (Fig. S2 and S3). Although minor differences in overall clade membership were observed between the MLST method and the two cg methods, the results were fairly consistent. Simpson’s diversity index was 0.987 for the cgMLST and 0.984 for the cgSNV analysis, indicating strong separation of the isolates into individual STs, with most isolates assigned a unique ST.

### wgSNV analysis.

**FIG 4:**
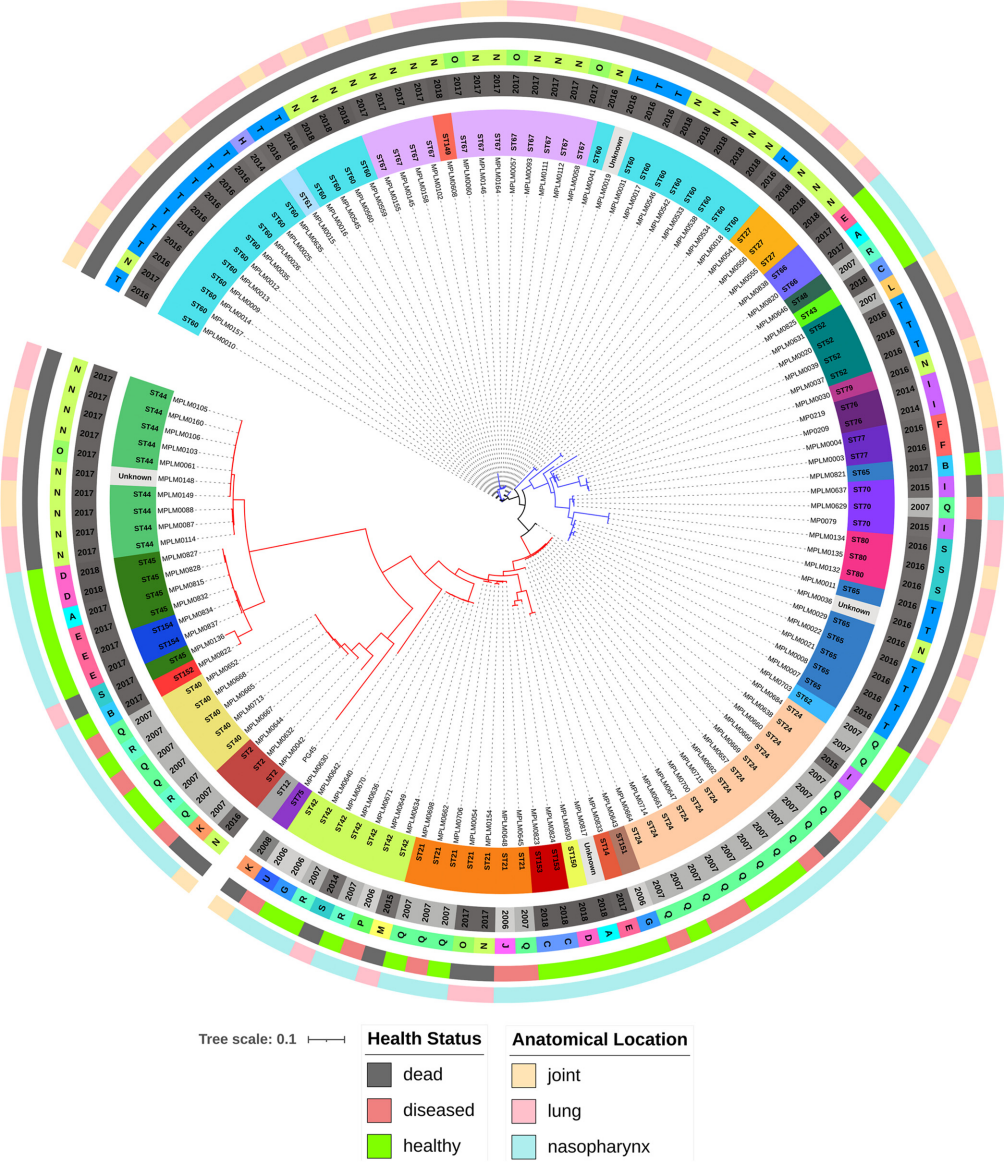
wgSNV maximum-likelihood tree of 130 Mycoplasma bovis genomes based on 14,383 SNVs in the core and accessory genomes. The rings (starting from the innermost) contain information on isolate identifiers, MLST sequence type (ST), production year, feedlot (A to U), anatomical sampling location, and animal health status at the time of sampling. Clades 1 and 2 are indicated by blue and red, respectively. Isolate MPLM0703 (ST62) falls evenly between these two clades and, as a result, was not included in either. These clades match the clades identified in Fig. S4.

All 130 genomes were typed by the wgSNV method, and no two isolates shared an identical SNV matrix; hence, each isolate was unique (Fig. 4; Fig. S4). As a result, Simpson’s diversity index was 1.000. Two primary clades (C1, C2) branched into two subclades of approximately equal size. A single isolate (ST62) was positioned evenly between the two clades. The wgSNV method was able to resolve isolates with the same MLST ST assignment as the cgMLST and cgSNV methods. Like the cgMLST and cgSNV methods, the wgSNV method grouped ST2, ST21, ST24, and ST42 in C1, while the ST70 isolates grouped with a separate lineage (C2) (Fig. S4).

## DISCUSSION

This is the first study to compare four different phylogenetic typing methods using a relatively large and diverse set of M. bovis isolates derived from western Canadian feedlot cattle. Overall, more genomes were typed by the MLST scheme (*n *= 126) than by the cgMLST or cgSNV method (*n *= 102), which required the assembly of hundreds of loci constituting a large portion of the genome. The wgSNV method successfully typed all 130 genomes, highlighting the robustness of the SPAdes and kSNP software, even with inputs of variable sequencing depth. All four methods had a high degree of discriminatory power, as judged by Simpson’s diversity index. MLST had the lowest discriminatory power, while cgMLST had a modestly higher index than the cgSNV method. Despite some differences in the phylogenetic outputs of the four methods, the MLST method generated a pattern of clonal complexes (CC) comparable to the clades generated by the cgMLST, cgSNV, and wgSNV methods. This is noteworthy, since the MLST scheme relied on seven housekeeping genes, representing <1% of the M. bovis PG45 reference genome. In contrast, the cgMLST scheme was derived from 506 loci, covering approximately 55% of the genome. The cgSNV method analyzed 3,925 SNVs from 283 loci, while the wgSNV matrix utilized 14,383 SNVs, generating the highest genotypic resolution.

Since core genome and whole-genome methods are based on a larger representation of the genome, theoretically they should have greater potential to resolve relationships than the seven-locus MLST scheme. This makes the cgMLST, cgSNV, and wgSNV methods ideally suited for epidemiological investigations where small differences between STs may be consequential. Parker et al. applied wgSNV analysis to 75 Australian M. bovis isolates and found a very high level of homogeneity among the isolates; the maximum number of single nucleotide polymorphisms (SNPs) between any two isolates was 50 (47). This level of resolution led the researchers to conclude that a single strain of M. bovis was circulating within Australia’s cattle population. This is quite unique, since a number of other country-level studies have found multiple clusters of genetically distant M. bovis STs within the cattle population (30–32, 48). Furthermore, these studies provided some insight into the movement of specific STs from country to country and over time. These higher-resolution methods have also been applied at the farm level, to examine transmission between cows and calves within the same dairy farm (29) and the introduction of M. bovis into dairy herds via contaminated semen (28).

The higher concordance and resolution of the cgMLST, cgSNV, and wgSNV methods were evident when the comparison focused on five STs (ST2, ST21, ST24, ST42, ST70) representing 33 isolates obtained between 2006 and 2017. The MLST scheme assigned three STs (ST2, ST21, ST24) to clonal complex 2 (CC2), ST70 to CC1, and ST42 as a singleton, whereas both the cgMLST and cgSNV analyses assigned four STs (ST2, ST21, ST24, ST42) to clade 3 (C3) and ST70 to C2. The wgSNV analysis yielded results similar to those of the cgMLST and cgSNV methods. These findings underscore that all four methods had similar assignments for four of the five STs. However, because ST42 was a triple locus variant, it became an outlier or singleton. Since the cgMLST, cgSNV, and wgSNV methods utilize hundreds to thousands of discrete data points to compare the genetic relatedness of isolates, they grouped ST42 alongside other STs into a clade.

The MLST method identified 30 different STs, underscoring the genetic diversity of M. bovis in western Canada. This is most likely related to the underlying structure of the Canadian cattle industry and the way in which feedlot cattle are procured. At the time of the last agricultural census (2016), Canada had approximately 54,000 beef cattle farms, 38,700 (72%) of which were located in western Canada (https://www150.statcan.gc.ca/n1/en/type/data?text=40221). Furthermore, western Canadian feedlots also import cattle from the United States. Since most feeder cattle are sold at auction, extensive commingling of cattle from multiple owners occurs during procurement. Once sold, the cattle are assembled, transported, and then processed and further commingled at the feedlot. This also occurs in the autumn months, when inclement weather conditions arise. Conceivably, commingling, transport, and changing environmental conditions all contribute to stress, which facilitates increased shedding from carrier animals (14). Thus, given the broad catchment from which cattle are sourced and mixed, it is understandable that feedlots had multiple STs circulating during the same time period. Interestingly, ST2 was isolated from two feedlots in 2007 (feedlots Q and K) and then, 9 years later, from feedlot N. Similarly, ST21 was isolated from feedlot J in 2006, from feedlot Q in 2007, and then from feedlots N and O in 2017. Not only were these isolates separated in time, but feedlots N and Q were located approximately 500 km apart. This separation by time and space suggests that some STs may be more dominant and widespread than others.

While Canada’s feedlot sector is concentrated in Alberta, there is bilateral trade in cattle between Canada and the United States, which is noteworthy because five isolates from American cattle were evenly distributed among the Canadian isolates within the NJ trees and MSTs (identified in Table S1 in the supplemental material). These results support the findings of a recent study in which wgSNV analysis found a high degree of genetic diversity among the American isolates, with Canadian isolates clustering within the same clade as the American isolates (32).

MLST identified 30 STs dispersed over 12 production years. Among these, two strains (ST21, ST52) had been reported in bovine isolates outside North America: ST21 was reported in Europe and Asia, whereas ST52 was reported in Europe, Asia, and Oceania (PubMLST isolate database [https://pubmlst.org/organisms/mycoplasma-bovis/], accessed 12 March 2021). This worldwide distribution underscores the international trade in cattle and the need for biosecurity measures to mitigate the transmission of M. bovis and other potentially production-limiting pathogens. It is also noteworthy that ST21 has also been isolated from bison (PubMLST isolate database, accessed 12 March 2021).

WGS allows typing by multiple methods to be done *in silico*, and Ridom Seqsphere+ makes high-resolution typing methods accessible to those without the knowledge required to construct a customized whole-genomic-analysis pipeline. Established typing methods, such as MLST, will invariably continue to support *in silico* efforts to conduct comparative studies using historical and contemporary data. WGS of isolates, particularly when analyzed with Ridom Seqsphere+, provides the opportunity to merge established methods (MLST) with more-robust core genome (i.e., cgMLST and cgSNV) approaches. Additionally, given that the cost of WGS is comparable to that of sequencing seven PCR amplicons, particularly for a small, 1-Mbp genome such as that of M. bovis, WGS is likely to become the standard for phylogenetic studies. Furthermore, the ability to generate high-quality M. bovis assemblies from long reads in a cost-effective manner will enable greater use of cgMLST, cgSNV, and wgSNV phylogenetic typing methods (49).

A caveat for using the cg methods is the need for greater sequencing depth and fewer sequencing artifacts in order to generate more-complete, higher-quality assemblies. This was evident from the fact that more isolates were typed by MLST than by the cgMLST or cgSNV method, while the wgSNV software pipeline was able to generate a phylogenetic tree for all isolates. The wgSNV approach was better able to process poorer-quality sequencing data because it analyzes the entire genome to a greater degree than the other methods. However, it is not without its weaknesses. The assembly software may have erroneously assembled the small subsets of the genome, resulting in false SNPs contributing to the uniqueness of the genotypes. However, misassemblies occur only infrequently and in sections of an assembly with poor coverage, making this occurrence in our data set unlikely. Care must also be taken in choosing analysis software and associated input parameters. This is exemplified in the NJ trees and MSTs, which were similar, but with differences in the positions of ST2, ST40, ST44, ST45, and ST75. This highlights the need for high-quality sequence data as the analysis moves from MLST to more-complex methods, such as the cgSNV or wgSNV method.

The design of this study was appropriate for comparing the four genotyping methods. However, the results were equivocal with regard to whether the lack of association between genotypes (STs) and phenotypes (year, health status, anatomical location) was real or was related to the limited number of isolates collected. Obtaining a complete set of DNP swabs, lung samples, and joint samples from each animal would have helped in determining whether STs exhibited a tropism for specific tissues. On this point, it was salient that the data set included paired lung-joint samples from 28 animals, 18 (64.3%) of which had the same ST in the lung and joint, suggesting the absence of single-tissue tropism. However, in 10 cases, the genotypes of lung and joint isolates within the same animal differed, underscoring the need for polyvalent vaccines. Others have also suggested that a polyvalent vaccine maybe required to cover the broad functional diversity of isolates (32).

The lack of association between genotype and phenotype is certainly not unique; rather, it is the emerging consensus. Multiple studies using high-resolution typing methods have been unable to show linkages between clusters and anatomical sample locations (47, 48) or health status (31, 47). However, one of the issues is that these studies have not been specifically designed to investigate these associations. This is problematic, since the lack of association may be related not only to an inadequate number of samples but also to an unbalanced study design. Many of the studies have biases in sampling related to year, anatomical location, geographical location, and health status. These confounding factors may result in type I and II errors. This is particularly true when one is investigating the association between genotype and health status in feedlot cattle. Animals deemed healthy on arrival and at the time of sampling may develop BRD within days. Conversely, BRD is a polymicrobial disease, and hence, clinical disease does not equate to mycoplasmosis. This conundrum is best addressed by comparing isolates from healthy animals with those obtained at the time of postmortem examination from tissues (lungs and joints) exhibiting pathology consistent with mycoplasmosis.

Each genotyping method has strengths and weaknesses depending on the research question. MLST is best suited for use as an initial screening method to detect the presence of genetically distinct strains and is amenable to both PCR and *in silico* methods. Furthermore, the M. bovis PubMLST database is curated and is accessible through a publicly available website. While cgMLST, cgSNV. and wgSNV analyses allow for the typing of strains, they also provide a higher level of genetic resolution, which may be used to discern clinically relevant differences, such as tissue tropisms, antimicrobial resistance, or virulence.

### Conclusion.

The wgSNV method successfully typed all 129 field isolates, whereas the cg methods typed 101 isolates, an outcome that may be rectified with greater sequencing depth. Overall, assignments of clade membership by MLST and the higher-resolution methods were similar on visual assessment of the NJ trees and MSTs; the cgMLST and cgSNV methods had the highest degree of concordance. The wgSNV approach provided an incrementally higher level of genomic resolution and detail, which may have utility in some epidemiological investigations and in addressing research questions relating to gene function and characterization. Although the wgSNV method was very powerful and robust, it is less user-friendly, requiring specialized training in bioinformatics. Conversely, the cg analyses were performed using Ridom SeqSphere+, which is graphical interface software that nonbioinformaticians with moderate background knowledge can use. Thus, it provides greater resolution than MLST and requires less specialized training, but it is relatively expensive if it is being used only on a limited number of isolates. None of the methods could show a clear association between genotype and phenotype, which may reflect the limitations of these methods or could be related to a relatively small sample size and inadequate study design.
