# Supplementary material for: Application of Four Genotyping Methods to Mycoplasma bovis Isolates Derived from Western Canadian Feedlot Cattle
Source: J Clin Microbiol. 2021 Jun 18;59(7):e00044-21. doi: 10.1128/JCM.00044-21 (PMC8218767; doi:10.1128/JCM.00044-21)
Supplement: Supplemental file 1 — . Download JCM.00044-21-s0001.pdf, PDF file, 997 KB [file jcm.00044-21-s0001.pdf]

Table S1 Isolate Data

Isolates previously submitted to pubMLST under a separate isolate ID are identified by that ID within the Alternate pubMLST ID and Notes column. Isolates with an Unknown ST have their missing allele(s) identified in this column, in addition to the five isolates that originate from the USA.

Sequence Type	Alternate pubMLST ID and Notes	ID	Year	Feedlot	Anatomical Location	Health Status	Host ID
2	MJ259	MPLM0042	2016	N	joint	dead	16
2		MPLM0632	2007	K	nasopharynx	dead	83
2		MPLM0644	2007	Q	nasopharynx	diseased	42
14	USA Isolate	MPLM0833	2017	E	nasopharynx	healthy	31
21	MJ260	MPLM0054	2017	O	lung	dead	70
21	MJ287	MPLM0154	2017	N	lung	dead	67
21		MPLM0645	2007	Q	nasopharynx	diseased	56
21		MPLM0648	2006	J	nasopharynx	diseased	73
21		MPLM0662	2007	Q	nasopharynx	diseased	51
21		MPLM0698	2007	Q	nasopharynx	healthy	59
21		MPLM0706	2007	Q	nasopharynx	healthy	55
24		MPLM0638	2015	I	joint	dead	88
24		MPLM0647	2007	Q	nasopharynx	diseased	45
24		MPLM0657	2007	Q	nasopharynx	diseased	46
24		MPLM0660	2007	Q	nasopharynx	diseased	40
24		MPLM0661	2007	Q	nasopharynx	diseased	44
24		MPLM0664	2007	Q	nasopharynx	diseased	39
24		MPLM0666	2007	Q	nasopharynx	diseased	57
24		MPLM0669	2007	Q	nasopharynx	dead	43
24		MPLM0684	2007	Q	nasopharynx	healthy	49
24		MPLM0692	2007	Q	nasopharynx	healthy	58
24		MPLM0700	2007	Q	nasopharynx	healthy	52
24		MPLM0714	2007	Q	nasopharynx	healthy	48
24		MPLM0715	2007	Q	nasopharynx	healthy	54
27		MPLM0541	2018	N	lung	dead	90
27		MPLM0555	2018	N	lung	dead	64
27		MPLM0556	2018	N	joint	dead	64
40		MPLM0652	2007	Q	nasopharynx	diseased	50

40		MPLM0665	2007	Q	nasopharynx	diseased	41
40		MPLM0667	2007	R	nasopharynx	healthy	78
40		MPLM0668	2007	R	nasopharynx	healthy	79
40		MPLM0713	2007	Q	nasopharynx	healthy	47
42		MPLM0634	2015	M	lung	dead	65
42	MJ292	MPLM0636	2014	S	lung	dead	97
42		MPLM0640	2006	G	nasopharynx	healthy	77
42		MPLM0642	2006	U	nasopharynx	diseased	71
42		MPLM0649	2006	P	nasopharynx	diseased	74
42		MPLM0670	2007	R	nasopharynx	healthy	75
42		MPLM0671	2007	R	nasopharynx	healthy	76
43		MPLM0825	2018	C	nasopharynx	healthy	26
44		MPLM0061	2017	O	joint	dead	69
44	MJ267	MPLM0087	2017	N	lung	dead	17
44	MJ268	MPLM0088	2017	N	joint	dead	17
44	MJ272	MPLM0103	2017	N	joint	dead	9
44	MJ273	MPLM0105	2017	N	lung	dead	35
44	MJ274	MPLM0106	2017	N	joint	dead	35
44	MJ278	MPLM0114	2017	N	lung	dead	8
44	MJ286	MPLM0149	2017	N	joint	dead	68
44	MJ289	MPLM0160	2017	N	lung	dead	12
45	MJ280	MPLM0136	2017	S	lung	dead	4
45		MPLM0815	2017	A	nasopharynx	healthy	19
45		MPLM0827	2018	D	nasopharynx	healthy	27
45		MPLM0828	2018	D	nasopharynx	healthy	28
45		MPLM0832	2017	E	nasopharynx	healthy	30
48		MPLM0646	2007	R	nasopharynx	healthy	80
52	MJ246	MPLM0020	2016	T	joint	dead	89
52	MJ255	MPLM0037	2016	T	lung	dead	60
52	MJ257	MPLM0039	2016	T	lung	dead	98
52		MPLM0631	2007	L	joint	dead	82
60	MJ237	MPLM0009	2016	T	lung	dead	10
60	MJ238	MPLM0010	2016	T	joint	dead	10
60	MJ240	MPLM0012	2016	T	joint	dead	95
60	MJ241	MPLM0013	2016	T	lung	dead	11
60	MJ242	MPLM0014	2016	T	joint	dead	11
60		MPLM0016	2016	T	joint	dead	62
60	MJ244	MPLM0017	2016	T	lung	dead	61
60	MJ245	MPLM0018	2016	T	joint	dead	61
60	MJ249	MPLM0025	2016	T	lung	dead	3
60	MJ250	MPLM0026	2016	T	joint	dead	3
60	MJ253	MPLM0031	2016	T	lung	dead	2

60		MPLM0035	2016	T	lung	dead	86
60	MJ258	MPLM0041	2016	N	lung	dead	16
60		MPLM0157	2017	N	lung	dead	87
60		MPLM0533	2018	N	lung	dead	7
60		MPLM0534	2018	N	joint	dead	7
60		MPLM0538	2018	N	joint	dead	14
60		MPLM0542	2018	N	joint	dead	90
60		MPLM0545	2018	N	lung	dead	91
60		MPLM0546	2018	N	joint	dead	91
60		MPLM0559	2018	N	lung	dead	93
60		MPLM0560	2018	N	joint	dead	93
60	MJ291	MPLM0635	2014	H	lung	dead	96
61	MJ243	MPLM0015	2016	T	lung	dead	62
62		MPLM0703	2007	Q	nasopharynx	healthy	53
65	MJ235	MPLM0007	2016	T	lung	dead	63
65	MJ236	MPLM0008	2016	T	joint	dead	63
65	MJ239	MPLM0011	2016	T	lung	dead	95
65	MJ247	MPLM0021	2016	T	lung	dead	6
65	MJ248	MPLM0022	2016	T	joint	dead	6
65	MJ251	MPLM0029	2016	N	lung	dead	1
65		MPLM0821	2017	B	nasopharynx	healthy	22
66		MPLM0820	2017	A	nasopharynx	healthy	21
66	USA Isolate	MPLM0838	2017	E	nasopharynx	healthy	34
67	MJ261	MPLM0057	2017	O	lung	dead	18
67	MJ262	MPLM0058	2017	O	joint	dead	18
67	MJ263	MPLM0060	2017	O	lung	dead	69
67	MJ269	MPLM0093	2017	N	lung	dead	13
67	MJ271	MPLM0102	2017	N	lung	dead	9
67	MJ276	MPLM0111	2017	N	lung	dead	5
67	MJ277	MPLM0112	2017	N	joint	dead	5
67	MJ282	MPLM0145	2017	N	lung	dead	66
67	MJ283	MPLM0146	2017	N	joint	dead	66
67		MPLM0155	2017	N	joint	dead	67
67	MJ288	MPLM0158	2017	N	joint	dead	87
67		MPLM0164	2017	N	joint	dead	36
70		MP0079	2015	I	lung	dead	88
70	MJ290	MPLM0629	2007	Q	nasopharynx	diseased	81
70	MJ293	MPLM0637	2015	I	lung	dead	37
75		MPLM0630	2008	K	joint	dead	84
76		MP0209	2014	I	joint	dead	85
76		MP0219	2014	I	lung	dead	85

77	MJ234	MPLM0003	2016	F	lung	dead	94
77		MPLM0004	2016	F	joint	dead	94
79	MJ252	MPLM0030	2016	N	joint	dead	1
80		MPLM0132	2016	S	lung	dead	38
80		MPLM0134	2016	S	lung	dead	15
80		MPLM0135	2016	S	joint	dead	15
149		MPLM0608	2018	N	lung	dead	92
150	USA Isolate	MPLM0830	2018	D	nasopharynx	healthy	29
151		MPLM0643	2006	G	nasopharynx	healthy	72
152		MPLM0822	2017	B	nasopharynx	healthy	23
153		MPLM0823	2018	C	nasopharynx	healthy	24
153		MPLM0824	2018	C	nasopharynx	healthy	25
154	USA Isolate	MPLM0834	2017	E	nasopharynx	healthy	32
154	USA Isolate	MPLM0837	2017	E	nasopharynx	healthy	33
Unknown	Missing dnaA, gltX, gpsA, gyrB, tdk	MPLM0019	2016	T	lung	dead	89
Unknown	MJ254, Missing gpsA	MPLM0036	2016	T	joint	dead	86
Unknown	MJ285, Missing gyrB	MPLM0148	2017	N	lung	dead	68
Unknown	Missing gpsA, Novel pta2	MPLM0817	2018	A	nasopharynx	healthy	20

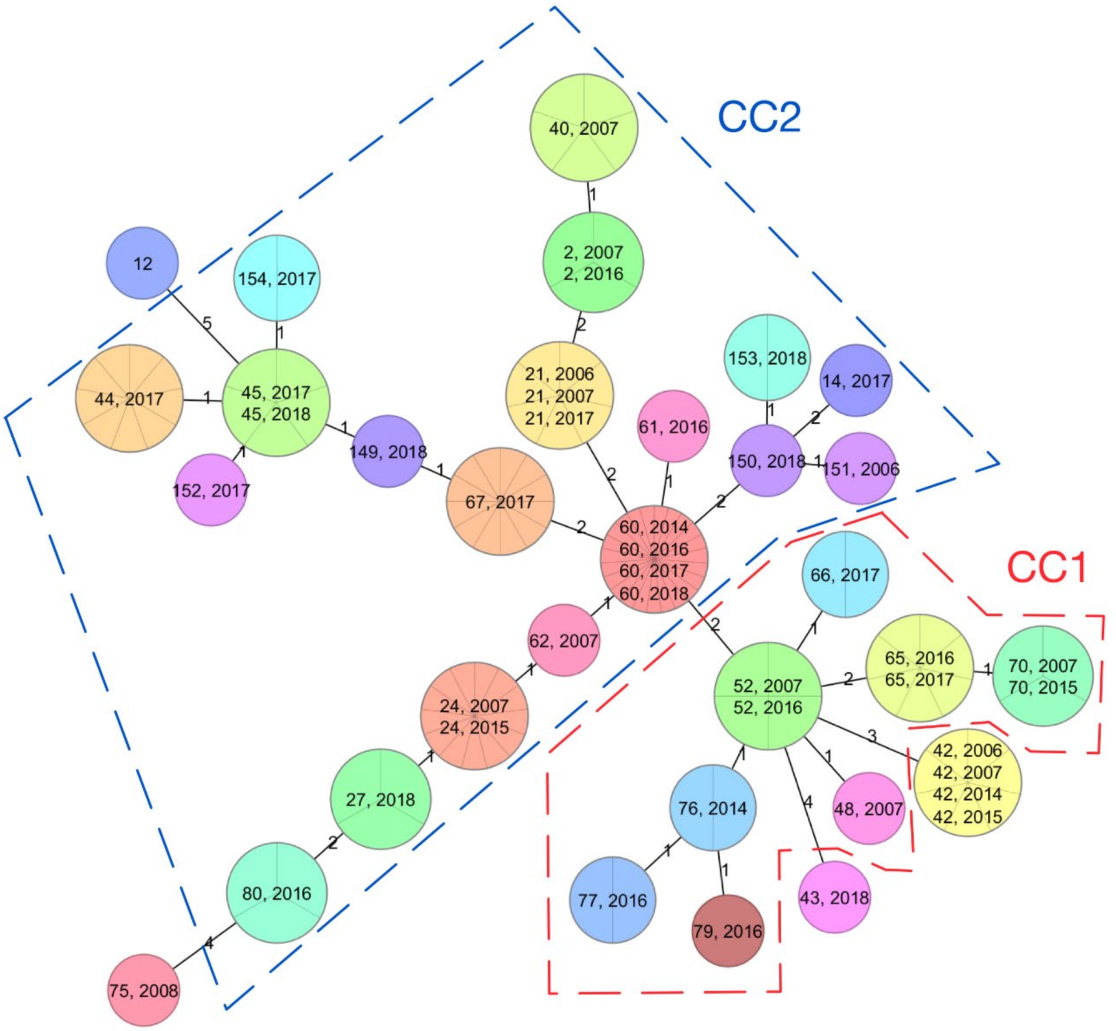

Figure S1 Minimum spanning tree of 126 *Mycoplasma bovis* genomes (125 field isolates plus PG45) typed by MLST. Clonal complexes 1 and 2 are surrounded by red and blue lines, respectively. Sequence types are colour coded with the size of the circle reflecting the number of isolates, with the partitioning lines within a circle delineating isolates with an identical genotype. The number of different alleles is indicated over the line connecting the sequence types. The PG45 reference genome is identified as the singleton with ST12.

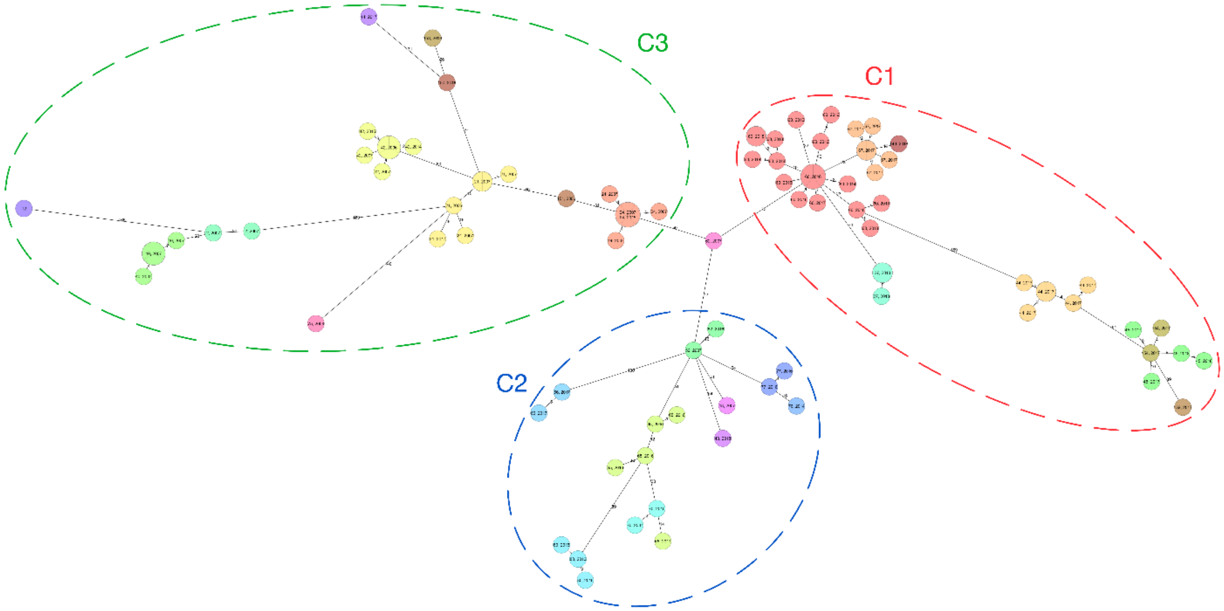

Figure S2 Minimum spanning tree of 102 *Mycoplasma bovis* isolates (101 field isolates plus PG45) created with cgMLST, based on alleles at 296 core genome loci. Clades 1 - 3 are denoted by a surrounding circle. The isolates are identified by MLST sequence type and production year (ST, Production Year). Sequence types are colour coded with the size of the circle reflecting the number of isolates, with the partitioning lines within a circle delineating isolates with an identical genotype. The number of different alleles is indicated over the line connecting the sequence types.

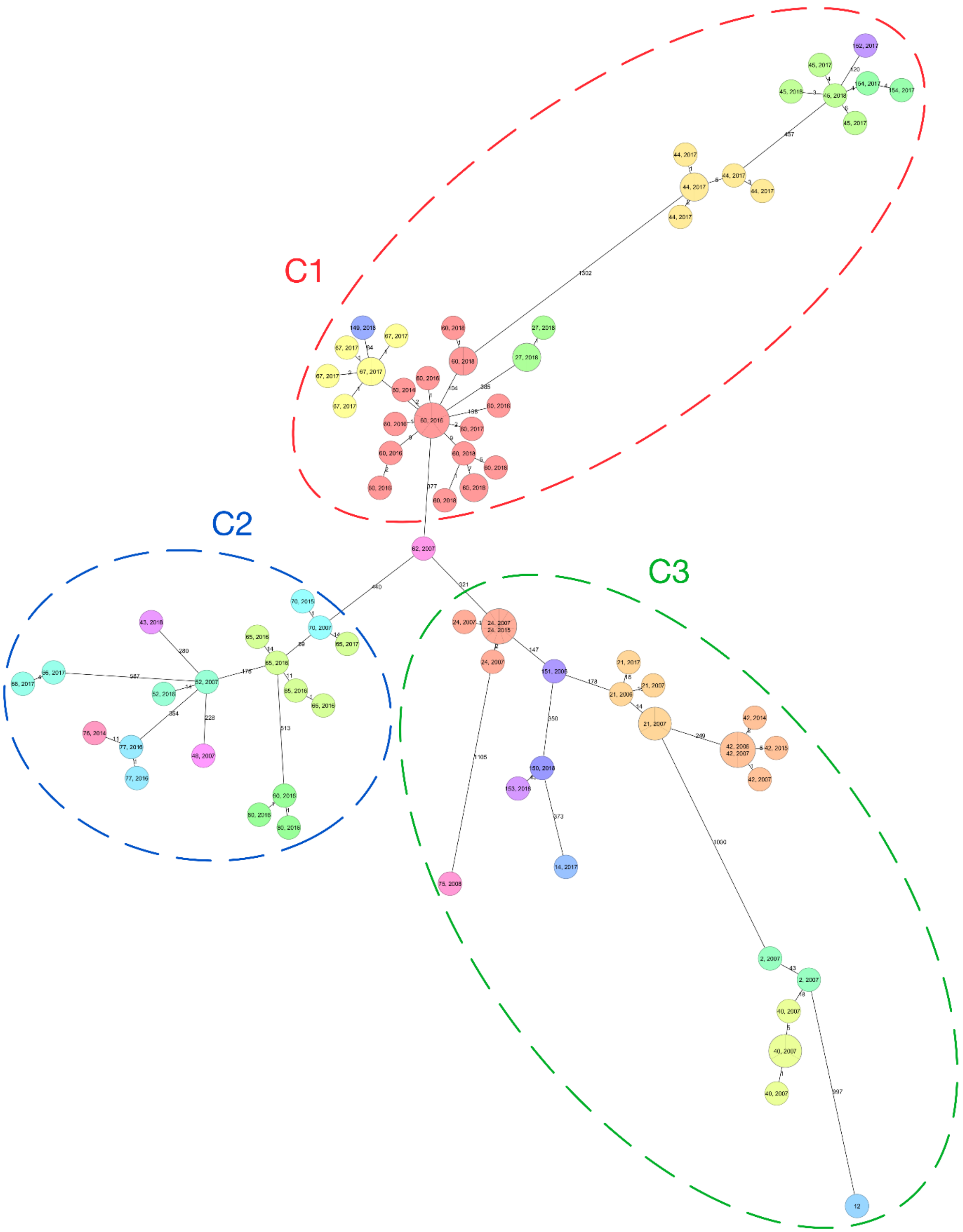

Figure S3 Minimum spanning tree of 102 *Mycoplasma bovis* isolates (101 field isolates plus PG45) based on 283 core genome loci, using cgSNV, based on 3,925 SNVs. The clades are

indicated by an encompassing circle. The 102 *M. bovis* isolates are identified by MLST sequence type and production year (ST, Production Year). The nodes are colour coded by the MLST sequence type. The size of the circle reflects the number of isolates represented, with the partitioning lines within a circle delineating isolates with an identical genotype. The number of different alleles is indicated over the line connecting the sequence types.

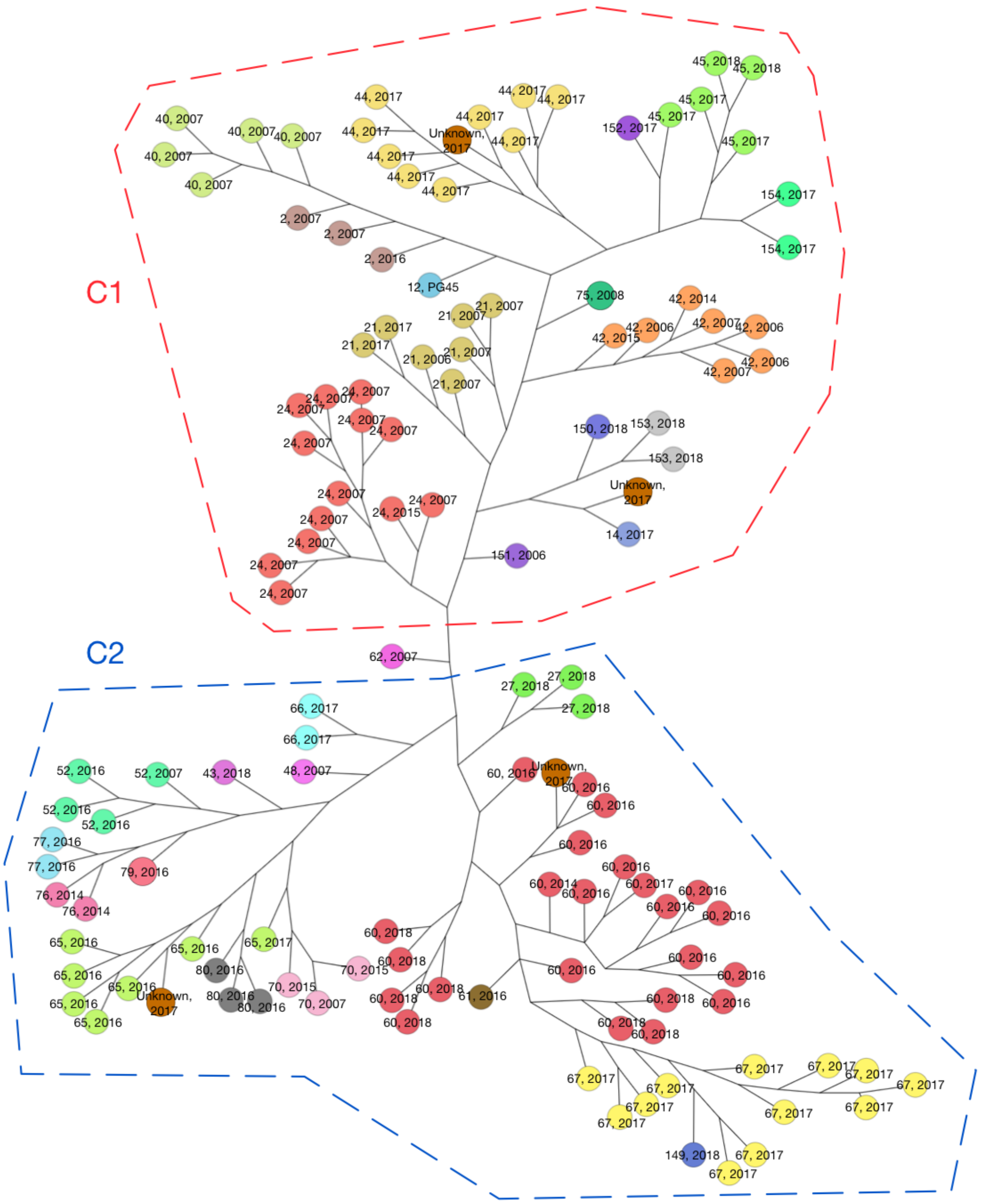

Figure S4 Maximum-likelihood tree of 130 *Mycoplasma bovis* isolates (129 field isolates plus PG45) based on 14383 SNVs in the core and accessory genomes typed by wgSNV. Clades 1 and 2 are indicated by a surrounding line in red and blue, respectively. The isolates are labelled with

MLST sequence type and production year (ST, Production Year), and colour coded with MLST sequence type. Each individual node represents a single isolate. No two isolates had identical SNV matrices.
